# Exploring the skin mycobiome in intensive care patients: a pilot study on fungal diversity from axillary and groin swabs

**DOI:** 10.1186/s12866-025-04288-7

**Published:** 2025-09-29

**Authors:** Teresa Nascimento, João Inácio, Daniela Guerreiro, Patrícia Patrício, Luís Proença, Cristina Toscano, Helena Barroso

**Affiliations:** 1 Egas Moniz Center for Interdisciplinary Research (CiiEM); Egas Moniz School of Health & Science, Caparica, Almada, 2829-511 Portugal; 2 School of Applied Sciences, University of Brighton, Brighton, UK; 3 Hospital Beatriz Ângelo, Loures, Portugal; 4 Centro Hospitalar Lisboa Ocidental Hospital Egas Moniz, Lisboa, Portugal; 5 Instituto Higiene e Medicina Tropical Universidade Nova de Lisboa, Lisboa, Portugal

**Keywords:** Mycobiome, Mycobiota, Skin, *Candida* spp., *Malassezia* spp., ICU.

## Abstract

The skin mycobiome is a largely unexplored component of the human microbiome, especially in critically ill patients. Fungal colonisation in the Intensive Care Unit (ICU) may be influenced by underlying comorbidities and hospital-related risk factors, potentially impacting patient outcomes. This pilot study aimed to characterize the diversity and abundance of skin fungi in ICU patients during their first week of hospitalization. A total of 35 ICU patients were recruited and divided into two groups: Group 1 (patients with 1–2 comorbidities and ICU risk factors) and Group 2 (patients with more than 2 comorbidities and ICU risk factors). Bilateral axillary-groin swabs were collected on admission day (D1) and after one week of ICU stay (D8) for fungal identification. Culture-based methods and MALDI-TOF MS were initially used, followed by Internal Transcribed Spacer 2 (ITS2) sequencing via the Illumina MiSeq platform for in-depth analysis of fungal communities. Fungal diversity and relative abundance were assessed using standard alpha and beta diversity metrics. Culture and MALDI-TOF MS identified only *Candida* spp., suggesting limited diversity. ITS2 sequencing revealed that *Candida* (69.3%) was the most prevalent genus, followed by *Penicillium* (10.3%) and *Cladosporium* (4.0%), with *Malassezia* being rare (0.7%). Diversity analysis indicated a relatively stable fungal community throughout the first week (ANOSIM, *p* = 0.499), with no significant changes in species richness or community structure. By Day 8, *Candida* spp. represented 79.9% and 78.9% of the mycobiome in Groups 1 and 2, respectively. Preliminary data suggest that the ICU skin mycobiome is dominated by *Candida* spp. and exhibits low fungal diversity. These findings provide foundational insight into the skin mycobiome of critically ill patients and underscore the need for further research to elucidate its clinical significance and potential role in patient management.

**Supplementary information:**

The online version contains supplementary material available at 10.1186/s12866-025-04288-7.

## Introduction

The skin serves as a protective barrier that hosts a complex and diverse array of microorganisms, including bacteria, fungi, archaea, and viruses, which vary across different skin regions based on each area’s unique microenvironment [1]. Fungi, as core component of the skin microbiome, contribute significantly to skin development, maintenance, and repair, highlighting the importance of studying skin fungal communities (mycobiomes) to better understand their roles in human health [2]. Mycobiomes are dynamic and vary by skin region and factors such as age and lifestyle [3–5].

Healthy skin is predominantly colonized by *Malassezia* spp., across all anatomical sites (face, arm, hand, axilla, inguinal region, back, scalp, and fingers of both hands and feet) [6]. These lipophilic yeasts are typically regarded as commensals, playing important roles in maintaining microbial homeostasis and supporting the skin barrier. However, environmental changes and medical interventions can disrupt this balance, increasing susceptibility to fungal overgrowth [3, 7]. Unlike *Malassezia*, which primarily acts as a commensal, *Candida* spp. are opportunistic yeasts with a greater tendency toward pathogenicity under immunosuppression or critical illness [8]. *Candida*’s potential for invasive growth makes it a major clinical concern in hospitals, particularly in immunocompromised patients [9].

Advancements in metagenomics have revealed greater fungal diversity than previously thought [10, 11]. Amplicon sequencing of the ITS region has been suggested as a reliable and relatively rapid method for analysing the mycobiome, being effective even in low biomass samples, as is common in skin samples [12, 13].

Intensive Care Unit (ICU) patients often face a high risk of fungal infections due to immunosuppression, invasive procedures, and broad-spectrum antibiotic use [14]. Such conditions often reduce microbial diversity, creating an environment where opportunistic pathogens like *Candida* spp. can thrive [10, 15].

This pattern suggests that similar dysbiotic shifts may occur across various microbial communities in ICU patients, including both bacterial and fungal populations but it is unclear if this microbial imbalance is a marker of the underlying disease, ICU treatment, or contributes to poor clinical outcomes [16].

In face of the above we aimed to conduct a preliminary investigation into the fungal diversity and composition of the ICU patients skin mycobiome, focusing on axillary and groin regions, and assess how ICU conditions influence fungal populations upon admission (D1) and on the eighth day (D8) of ICU stay.

## Materials and methods

### Study design and setting

This was a prospective, observational pilot study conducted at Beatriz Ângelo Hospital (BAH); a 424-bed suburban hospital located in the Lisbon metropolitan area. The hospital includes a 22-bed general Intensive Care Unit (ICU) and handles an average of approximately 330 admissions per day. The study aimed to evaluate the feasibility of skin mycobiome sampling and assess preliminary trends in fungal colonisation among critically ill patients. Fungal colonisation was defined by the presence of fungal organisms on the skin, as detected either by culture or molecular methods, regardless of clinical signs of infection.

### Patient population and eligibility criteria

A total of 35 adult ICU patients were enrolled and sampled at two time points: upon admission (Day 1, D1) and on the eighth day (D8) of ICU stay. Inclusion criteria were as follows: (1) age ≥ 18 years; (2) non-pregnant female or male patients; (3) capacity to provide informed consent (or surrogate consent, when applicable); (4) expected ICU stay of ≥ 7 days; and (5) presence of two or more comorbidities or recognized risk factors for fungal colonisation or infection, including diabetes mellitus, chronic kidney disease, chronic pulmonary disease, recent or ongoing antibiotic therapy, use of central venous catheters, mechanical ventilation, or urinary bladder catheterization. Patients were included irrespective of their primary diagnosis, with the objective of capturing a heterogeneous ICU population under routine care. This pilot study was not statistically powered to test specific hypotheses but was instead designed to explore sampling feasibility, generate preliminary data, and inform the design of future large-scale studies focused on the skin mycobiome in critical illness.

Patients were assigned to one of the groups based on underlying conditions and ICU risk factors commonly associated with fungal infections and most prevalent within our cohort: Group 1 (patients with 1–2 comorbidities and ICU risk factors; 16 out of 35, 46%) and Group 2 (patients > 2 comorbidities and ICU risk factors; 19 out of 35; 54%). Baseline data from both groups at D1 served as a comparator for evaluating the impact of ICU-related factors on the skin fungal microbiota following one week in the ICU (D8).

As part of the hospital’s infection control protocol, all patients were bathed daily with a solution containing 4% (v/v) chlorhexidine gluconate (CHG) upon admission until day five of their stay in the ICU. Swab samples collected on admission day (D1) were obtained after the first CHG bath. From day six onward, daily hygiene was performed using standard soap and water, in accordance with routine care procedures. Participation was voluntary and authorized through informed consent, signed either by the patient or an authorized representative. This study was approved by Hospital Ethics Committee.

### Combined axilla-groin swab sample

Bilateral combined axilla/groin swabs were collected using the Σ-transwab^®^ system transport (Sigma Transwab – Liquid Amies) at two time points: day 1 (D1) upon ICU admission and day 8 (D8) of hospitalization. A total of 70 samples were obtained, with 35 collected on D1 and 35 on D8, corresponding to the two study groups. Swab sampling was performed by trained clinical personnel following a standardized internal Standard Operating Procedure (SOP) to ensure methodological consistency and reproducibility. Each swab was applied to approximately 2–3 cm² of intact, non-damaged skin in both the axillary and groin regions. The swab was gently rubbed back and forth across the skin surface ten times with moderate, consistent pressure, to optimize fungal collection while minimizing the risk of irritation or abrasion. Swabs were never applied to damaged, inflamed, or ulcerated skin, both to ensure patient safety and to avoid introducing confounding microbial communities.

### Mycological cultures

**Fig. 1:**
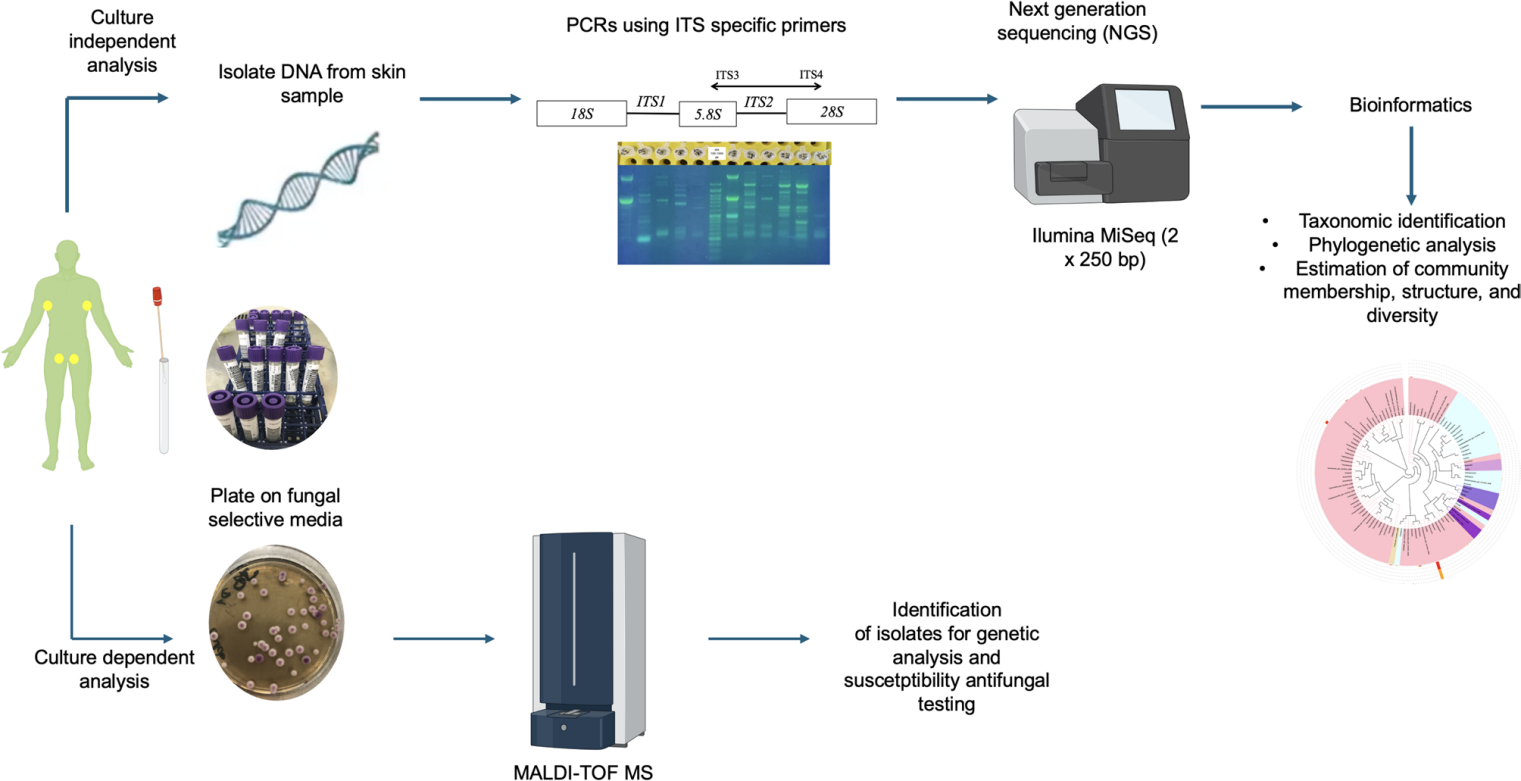
Flowchart of Mycobiome Study Protocols: Integrating Culture-Dependent and Culture-Independent Analyses (created with www.Biorender.com) (Supplementary file: Fig. S1)

Swabs were vortexed for 30 s, and 50 µl was transferred onto Sabouraud Gentamicin Chloramphenicol 2 agar (SGC2) (bioMérieux, Marcy l’Etoile, France) and a commercial *Candida* Chromogenic Medium (CHROMagar™ Candida, Paris, France). The plates were then aerobically incubated in two sets of different temperatures, 35 °C and 25 °C, for 48 h. After incubation, colonies were counted and classified by their colour. Further identification was performed using MALDI-TOF Vitek MS^®^ v3.2 mass spectrometry (bioMérieux, Marcy l’Etoile, France) (Fig. 1).

### DNA extraction and sequencing

#### DNA extraction

Swabs aliquots of 100 µl were used for total DNA extraction with the NZY Soil gDNA Isolation^®^ kit (NZYTech, Lisbon, Portugal). This kit, which includes a bead-beating step, was employed according to the manufacturer’s instructions. To confirm the quantity and quality of the DNA sample, a pan fungal PCR was carried out. The PCR reaction was optimized from the one described by Lau et al., 2007 [17]. To minimize contamination in low-biomass skin samples, negative controls were included during DNA extraction and PCR. Extraction blanks and no-template PCR controls were processed alongside samples and showed no amplification, confirming absence of contamination. Samples were stored at −20 °C. Metagenome sequencing was performed at the Novogene UK Cambridge Sequencing Center (Fig. 1).

#### Library preparation and sequencing

The concentration of the extracted genomic DNA was measured using Qubit 2.0 Fluorometer (Life Technologies), and the quality of the DNA was verified through gel electrophoresis. PCR reactions were performed with 10 ng of DNA using primer sets specific to the hypervariable regions ITS3-2024 F (5′GGAAGTAAAAGTCGTAACAAGG-3′) and ITS4-2409R (5′GCTGCGTTCTTCATCGATGC-3′). Each primer set included unique barcodes. PCR products were separated on gels, and fragments of the correct amplification size were extracted and purified. These PCR products were pooled in equal amounts, then, mixture PCR products were purified with Universal DNA Purification Kit (TianGen, China, Catalog #: DP214). The purified PCR products were then used as templates for library preparation. Sequencing libraries were generated using NEB Next^®^ UltraTM II FS DNA PCR- free Library Prep Kit (New England Biolabs, USA, Catalog #: E7430L) following manufacturer’s recommendations and indexes were added. The library was checked with Qubit and real-time PCR for quantification and bioanalyzer for size distribution detection. Quantified libraries were pooled and sequenced on Illumina Miseq^®^ (2 × 250 bp) (Illumina, San Diego, CA, USA) (Fig. 1).

### Bioinformatics analysis pipeline

#### Paired end reads assembly and quality control

Paired end reads were assigned to samples based on their unique barcodes and.

truncated by cutting off the barcodes and primer sequences. The whole process was performed through Python (v3.6.13) and adaptors were removed through Cutadapt (v3.3). For sequence assembly paired-end reads were merged using FLASH (v1.2.11, http://ccb.jhu.edu/software/FLASH/) [18]. Quality filtering on the raw tags were performed using the Fastp (v 0.23.1) software to obtain high-quality clean tags [19]. The tags were compared with the reference database (Unite Database (ITS), https://unite.ut.ee/) using UCHIME Algorithm (http://www.drive5.com/usearch/manual/uchime_algo.html) to detect chimera sequences. The effective tags were obtained by removing the chimera sequences with the VSEARCH (v2.16.0) package [20]. For each representative sequence, the Unite Database (https://unite.ut.ee/) [21] was used based on blast algorithm to annotate taxonomic information.

#### Operational taxonomic units cluster and species annotation

Sequence analyses were performed by Uparse software (Uparse v7.0. 1001,

http://drive5.com/uparse/) [20]. For each representative sequence, the Unite Database (https://unite.ut.ee/) [21] was used based on blast algorithm to annotate taxonomic information. To study phylogenetic relationship of different Operational Taxonomic Units (OTUs), and the difference of the dominant species in different samples (groups), multiple sequence alignment was conducted using the MUSCLE software (Version 3.8.31http://www.drive5.com/muscle/) [22].

The number of read counts for each taxon identified per sample was used to build an abundance table comprising information from all samples. The abundance table was used for composition, alpha and beta diversities and differential abundance analyses (Supplementary file: Fig. S2). OTUs abundance information were normalized using a standard of sequence number corresponding to the sample with the least sequences. Subsequent analysis of alpha diversity and beta diversity were all performed basing on this output normalized data.

#### Diversity analysis

Alpha and beta diversity calculations were performed using the QIIME (Version 1.9.1) and displayed with R software (Version 4.0.3). The alpha diversity indices of fungal communities were calculated using Observed-species, Chao1, Shannon and Simpson indices (Supplementary file: Fig. S3). The number of reads selected for normalization was set at a cutoff of 27,255. To evaluate the richness of microbial community and sample size, species accumulation boxplots were used to visualize, which were performed with vegan package 2.6-4 in R software (Version 4.0.3).

Cluster analysis for beta diversity was preceded by principal component analysis (PCA), which was applied to reduce the dimension of the original variables (Supplementary file: Fig. S4). Beta diversity was analysed with Principal Coordinates Analysis (PCoA) to get principal coordinates and visualize from complex, multidimensional data. PCA and PCoA analysis was displayed by ade4 package and ggplot2 package [23] in R software (Version 4.0.3). To study the genetic proximity between the different samples, cluster analysis was used to build a phylogenetic tree. The Unweighted Pair-group Method with Arithmetic Mean (UPGMA) was used.

### Statistical analysis

Data analysis was carried out by using descriptive and inferential methodologies using the IBM SPSS Statistics v. 29.0 (IBM Corp., Armonk, NY) software. For all the inferential analyses, a *p*-value of less than 0.05 was considered statistically significant.

The Student t-test was performed to determine whether the UniFrac dissimilarities, alpha diversities, and taxonomies of the two groups (D1 and D8) were significantly different. A series of statistical analyses which include ANOSIM and Adonis, were conducted to identify the factors that differentiate the microbial community. All of them were performed with vegan package 2.6-4 and ggplot2 package 3.4.0 within R software (Version 4.0.3).

## Results

### Clinical and demographic profile of ICU patient’s cohort

We enrolled 35 patients with an 8-day length of stay in the ICU at *Beatriz Ângelo Hospital* (Lisbon, Portugal). No patients exhibited signs of fungal infection during the recruitment period based on standard clinical diagnostic criteria. Out of the 70 samples collected, PCR amplification of the ITS genes was successful in 56 samples. Of these, 42 samples were from 21 patients, collected on both day 1 (D1) and day 8 (D8). Additionally, 8 samples were collected only on D1 and 6 only on D8.

Among this cohort, 71.4% were males. The average age was 65 years, with a range from 39 to 90 years. The average APACHE score was 13. The complete clinical characterization of the cohort is described in Table 1.

**Table 1:** Demographics and clinical characteristics of the patients cohort^a^

Patient characteristics	Group 1 (1–2 risk factors) *n* = 16	Group 2 (> 2 risk factors) *n* = 19	Cohort *n* = 35	*p*
Age – year – median	65.5	65.0	65	0.637
18–50 year	4 (25.0)	4 (21.1)	8 (22.8)	
51–80 year	12 (75.0)	14 (73.7)	26 (74.3)	
> 80 year	0 (0.0)	1 (5.3)	1 (2.9)	
Sex				0.283
Male	10 (62.5)	15 (78.9)	25 (71.4)	
Female	6 (37.5)	4 (21.1)	10 (28.6)	
ICU hospitalization secondary to				
Pulmonary infection	1 (6.3)	2 (10.5)	3 (8.6)	0.653
Cardiovascular disease	6 (37.5)	4 (21.1)	10 (28.6)	0.283
Gastrointestinal pathology	0 (0.0)	7 (36.8)	7 (20.0)	0.007
Urinary tract infection	0 (0.0)	3 (15.8)	3 (8.6)	0.096
COVID-19	2 (12.5)	0 (0.0)	2 (5.7)	0.112
APACHE– median	11.5	13.5	13	0.574
Risk factors				
Presence of CVC^b^	1 (6.3)	13 (68.4)	14 (40.0)	< 0.001
Mechanical ventilation	0 (0.0)	7 (36.8)	7 (20.0)	0.007
TPN^c^	0 (0.0)	1 (5.3)	1 (2.9)	0.352
Abdominal surgery	0 (0.0)	10 (52.6)	10 (28.6)	< 0.001
Neutropenia	0 (0.0)	1 (5.3)	1 (2.9)	0.352
Vesical catheter	4 (25.0)	13 (68.4)	17 (48.6)	0.010
Dialysis	0 (0.0)	1 (5.3)	1 (2.8)	0.352
Solid tumour	0 (0.0)	9 (47.4)	9 (25.7)	0.001
Hematological neoplasms	0 (0.0)	1 (5.3)	1 (2.9)	0.352
Diabetes *mellitus*	3 (18.8)	7 (36.8)	10 (28.6)	0.238
HIV/AIDS	0 (0.0)	2 (10.5)	2 (5.7)	0.181
Anemia (Hb < 10 mg/dl)	0 (0.0)	2 (10.5)	2 (5.7)	0.181
Severe immunodeficiency	0 (0.0)	1 (5.3)	1 (2.9)	0.352
Skin damage				0.112
Yes	2 (12.5)	0 (0.0)	2 (5.7)	
No	14 (87.5)	19 (100.0)	33 (94.3)	
Antibiotics exposure^d^				0.713
Beta-lactams	3 (18.8)	9 (47.4)	12 (34.3)	
Macrolides	2 (12.5)	0 (0.0)	2 (5.7)	
Fluoroquinolones	1 (6.3)	1 (5.3)	2 (5.7)	
Tetracycline	0 (0.0)	0 (0.0)	7 (20.0)	
Aminoglycoside	0 (0.0)	5 (100.0)	5 (14.3)	
Glycopeptide	0 (0.0)	7 (100.0)	7 (20.0)	
Others	0 (0.0)	2 (100.0)	2 (5.7)	
Antifungal therapy	0 (0.0)	0 (0.0)	0 (0.0)	NA
3 Months Outcome				0.060
Alive	11 (68.8)	7 (36.8)	18 (51.4)	
Deceased	5 (31.3)	12 (63.2)	17 (48.6)	

^a^Data are presented as No. (%) unless otherwise specified

^b^Central Venous Catheter (CVC)

^c^Total parental Nutrition (TPN)

^d^48 h prior. Not appliable (NA)

### Fungal identification by culture-dependent methods

To assess viable fungal colonisation, culture-based methods were employed using standard microbiological procedures. Swabs collected from the bilateral axillary-groin region of ICU patients were inoculated onto selective fungal media (SGC2) and chromogenic *Candida* medium (CHROMagar), then incubated under conditions optimized for yeast growth. Isolated colonies from both media were identified to the species level using MALDI-TOF MS. The cohort included 20 patients (S14-S175) from 2021 to 15 patients (S177-S491) from 2022.

Within this cohort, fungal cultures yielded exclusively species from the genus *Candida*, predominantly *C. albicans* and *C. parapsilosis* complex. In total, *Candida* species were detected in 48 out of 56 processed samples, representing 31 individual patients. Among the positive samples, the most frequently identified species were *C. albicans*, followed by *C. parapsilosis*, *C. tropicalis*, and *C. glabrata*.

The majority of positive cultures (44 out of 48) yielded a single *Candida* species per sample, whereas a few samples showed mixed colonisation with two *Candida* species.

For four patients (six samples), no fungal growth was detected. In two additional cases, only one of the two time-point samples per patient was culture-positive (e.g., S83D8 and S366D8). A full list of species and their distribution across patient samples is summarized in Table 2.

**Table 2:** *Candida* species detected by culturing or MALDI-TOF MS and ITS2 sequencing

Source^a^	Candida species detected by culture-based methods	Candida species detected by sequencing
S14.D1/D8	Negative	*C. albicans*,* C. parapsilosis*,* C. lusitaniae*,* C. glabrata*
S15.D8	*C. glabrata*	*C. albicans*,* C. parapsilosis*,* C. glabrata*,* C. lusitaniae*
S30.D1/D8	*C. parapsilosis*	*C. albicans*,* C. parapsilosis*,* C. glabrata*,* C. lusitaniae*
S31.D1/D8	*C. albicans*,* C. parapsilosis*	*C. albicans*,* C. parapsilosis*,* C. tropicalis*, *C. glabrata*,* C. lusitaniae*
S32.D1	*C. parapsilosis*	*C. albicans*,* C. parapsilosis*,* C. tropicalis*
S33.D1	Negative	*C. albicans*,* C. parapsilosis*
S38.D1^b^/D8^c^	*C. albicans*,* C. parapsilosis*	*C. albicans*,* C. parapsilosis*,* C. tropicalis*
S39.D1^c^/D8^c^	*C. albicans*,* C. parapsilosis*	*C. albicans*,* C. parapsilosis*,* C. tropicalis*, *C. glabrata*,* C. lusitaniae*
S43.D1/D8	*C. albicans*	*C. albicans*,* C. parapsilosis*,* C. tropicalis*, *C. glabrata*,* C. lusitaniae*
S52.D1/D8	*C. albicans*	*C. albicans*,* C. parapsilosis*,* C. tropicalis*, *C. lusitaniae*
S57.D1/D8	*C. parapsilosis*,* C. tropicalis*	*C. albicans*,* C. parapsilosis*,* C. tropicalis*, *C. lusitaniae*
S68.D1/D8	*C. albicans*	*C. albicans*,* C. parapsilosis*,* C. tropicalis*, *C. sake*
S79.D1/D8	*C. albicans*	*C. albicans*,* C. parapsilosis*,* C. tropicalis*, *C. glabrata*,* C. lusitaniae*
S83.D1^d﻿^/D8	*C. albicans*	*C. albicans*,* C. parapsilosis*,* C. tropicalis*, *C. lusitaniae*
S88.D1	*C. albicans*	*C. albicans*,* C. parapsilosis*,* C. tropicalis*, *C. glabrata*,* C. lusitaniae*, *C. guilliermondii*
S110.D1/D8	*C. albicans*	*C. albicans*,* C. parapsilosis*,* C. tropicalis*, *C. glabrata*,* C. lusitaniae*
S116.D1/D8	*C. parapsilosis*	*C. albicans*,* C. parapsilosis*,* C. tropicalis*, *C. glabrata*,* C. lusitaniae*
S171.D1	*C. tropicalis*	*C. albicans*,* C. parapsilosis*,* C. tropicalis*, *C. glabrata*,* C. lusitaniae*, *C. guilliermondii*
S175.D8	*C. albicans*	*C. albicans*,* C. parapsilosis*,* C. tropicalis*, *C. glabrata*
S177.D1/D8	*C. albicans*	*C. albicans*,* C. parapsilosis*,* C. tropicalis*, *C. glabrata*,* C. lusitaniae*
S196.D8	*C. albicans*	*C. albicans*,* C. parapsilosis*,* C. tropicalis*, *C. glabrata*,* C. lusitaniae*
S256.D1	*C. albicans*	*C. albicans*,* C. parapsilosis*,* C. tropicalis*, *C. lusitaniae*
S270.D1^b^/D8^c﻿^	*C. albicans*,* C. parapsilosis*	*C. albicans*,* C. parapsilosis*
S314.D1/D8	*C. albicans*	*C. albicans*,* C. parapsilosis*,* C. tropicalis*, *C. glabrata*,* C. lusitaniae*
S366.D1^d^/D8	*C. albicans*	*C. albicans*,* C. parapsilosis*,* C. tropicalis*, *C. glabrata*,* C. lusitaniae*, *C. guilliermondii*
S369.D1	*C. albicans*	*C. albicans*,* C. parapsilosis*
S406.D1/D8	*C. albicans*	*C. albicans*,* C. parapsilosis*
S422.D1/D8	Negative	*C. albicans*,* C. parapsilosis*
S425.D8	Negative	*C. albicans*,* C. parapsilosis*,* C. tropicalis*, *C. lusitaniae*
S426.D1/D8	*C. albicans*	*C. albicans*,* C. parapsilosis*
S431.D1	*C. albicans*,* C. parapsilosis*	*C. albicans*,* C. parapsilosis*
S442.D8	*C. albicans*	*C. albicans*,* C. parapsilosis*
S483.D8	*C. albicans*	*C. albicans*,* C. parapsilosis*, *C. lusitaniae*
S487.D1	*C. parapsilosis*	*C. albicans*,* C. parapsilosis*,* C. tropicalis*
S491.D1/D8	*C. parapsilosis*	*C. albicans*,* C. parapsilosis*

^a^Combined axillary/inguinal swab

^b^Only *C. albicans* was retrieved

^c^Presence of a mixed culture with *C. albicans*

^d^Positive culture for only one of the samples

### Sequence preprocessing

We built ribosomal DNA internal transcribed spacer 2 (ITS2) libraries for estimating fungal genera and species relative abundance in 56 samples. We generated a total of 4,115,291 raw reads (mean ± SD: 73,487 ± 24,481). For clean reads —high-quality sequences free from adapters, low-quality bases, and contaminants the total count was 72,349 ± 23,844, with a minimum count of 31,796. The number of non-chimeric reads, referring to sequences with chimeric artifacts removed, averaged 70,722 ± 23,842, with a minimum of 27,911.

The taxonomic assignment was conducted using the 97% identity for the UNITE (v9.0) database. Operational Taxonomic Units (OTUs) were estimated using QIIME1 (v1.9.1), resulting in 425 fungal genera and 577 fungal species, with an average of 80 taxa identified per sample (range 5 to 463).

### Relative abundance at the taxonomic level of phylum, genus and species

**Fig. 2:**
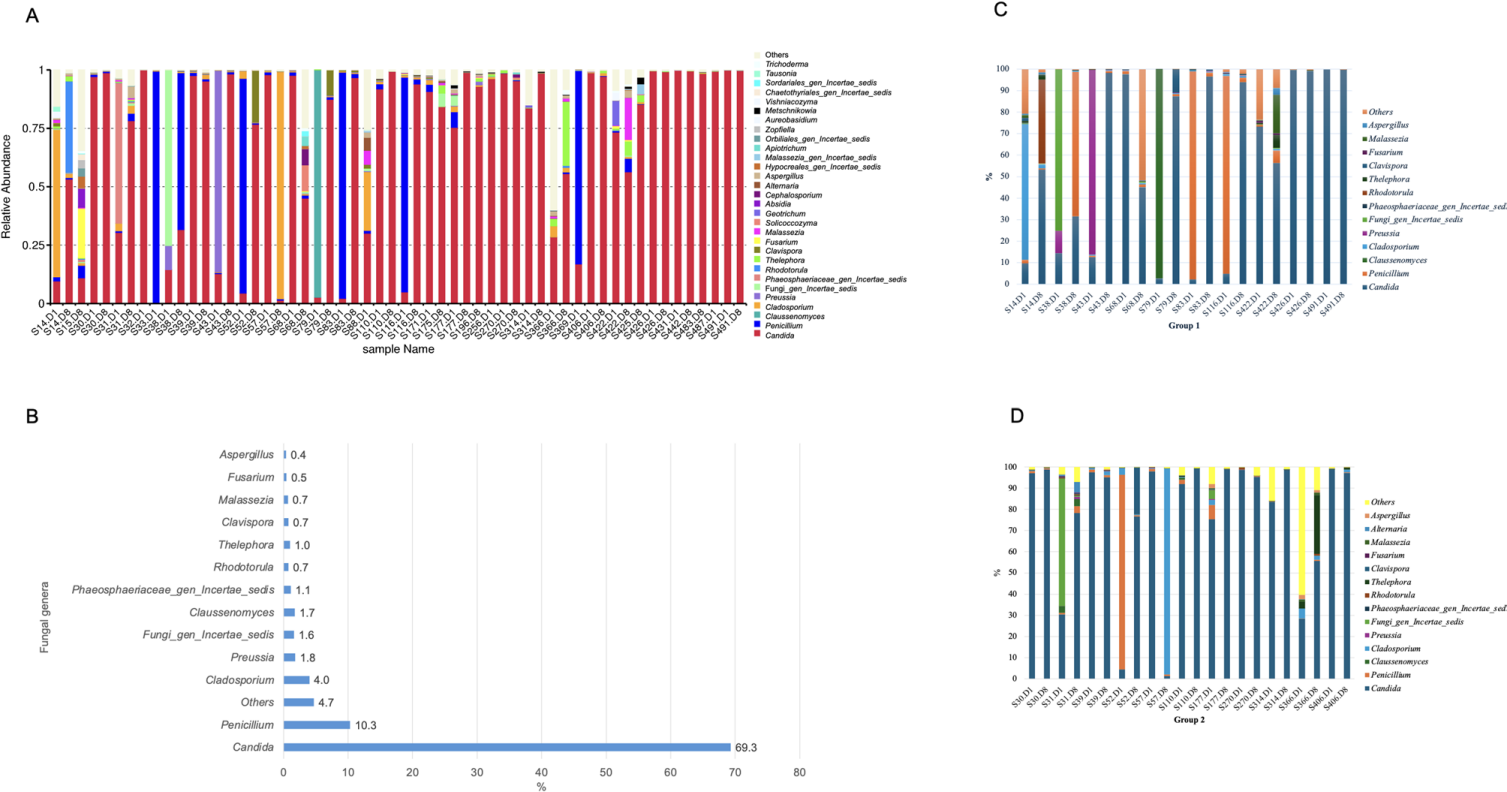
**A** Relative abundance of the 30 most prevalent fungal genera detected by culture-independent sequencing analyses in each individual skin swab sample. Bars are ordered by participant ID, which reflects the chronological order of prospective sample collection (corresponding to ICU admission dates). **B** Median relative abundance (%) of these fungal genera across all 56 samples. **C** Relative abundance of the most prevalent fungal genera detected threshold 0.4% for Group 1 paired samples. **D** Relative abundance of the most prevalent fungal genera detected threshold 0.4% for Group 2 paired samples. “Others” indicates the cumulative relative abundance of all remaining genera not individually shown

The composition of the mycobiome at the taxonomic level of the phylum revealed that 99.6% of the mycobiome belongs to the phylum Ascomycota, with only 0.4% belonging to the phylum Basidiomycota. According to the results of the taxonomic annotation, the relative abundance of 30 main fungal genera for each sample is illustrated, in Fig. 2A. The *Candida* genus is represented in all but one of the samples.

When the average relative abundance of the fungal genera present in the mycobiome of all the samples is considered, diversity is not high at values above 0.4%, with 69.3% of the mycobiome consisting exclusively of *Candida* species (Fig. 2B). Regarding the diversity of species of *Candida* spp. above 1.5%, only the genomes of *C. albicans* (34.2%), *C. parapsilosis* (33.3%) and *C. tropicalis* (1.6%) were identified. However, for lower abundance, *C. lusitaniae*, *C. glabrata*, *C. guilliermondii* and *C. sake* were also identified. For *Malassezia* species, all were detected less than 1.5% and included *M. restricta*, *M. globosa*, *M. sympodialis*, *M. arunalokei* and *M. dermatis*.

To further explore differences within the entire mycobiome cohort, the relative abundance of the most prevalent fungal genera (threshold ≥ 0.4%) was analysed separately for paired samples from patients in Group 1 (1–2 risk factors, *n* = 10) and Group 2 (> 2 risk factors, *n* = 11). These subgroup results are presented in Fig. 2C (Group 1) and Fig. 2D (Group 2). This stratification highlights the predominance of *Candida* across both groups, while also revealing subtle variations in the abundance of other fungal genera.

### Mycobiome diversity and community structure at day 1 and day 8

Regarding the relative abundance of the species, globally, *C. albicans* and *C. parapsilosis* co-occur in 93% of the samples. In D8, the most prevalent fungal species is *C. albicans* followed by *C. parapsilosis*, both as dominant species in a mycobiome with low diversity.

**Fig. 3:**
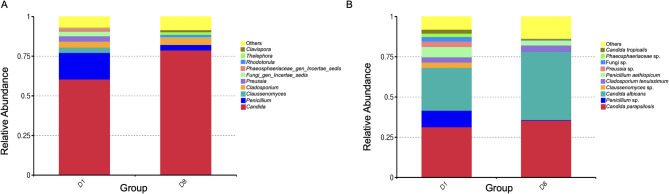
**A** Relative abundance of the 10 main fungal genera on D1 and D8 in the ICU. **B** Relative abundance of the 10 main fungal species on D1 and D8 in the ICU. “Others” represents the total relative abundance of species other than those named

When comparing the mycobiome on admission (D1) and after 8 days in the ICU (D8), an increase in the relative abundance of *Candida* spp. and a decrease in *Penicillium* spp. were observed (Fig. 3A). The variation in the predominant species between the two groups (D1 and D8) showed an increase in *C. albicans* followed by *C. parapsilosis*. Conversely, a decline in *C. tropicalis* was observed. It should be noted that in the D8 group, the mycobiome is mostly represented by only six fungal species (Fig. 3B).

### Alpha and beta diversity indices in for samples collected on day 1 and day 8

**Fig. 4:**
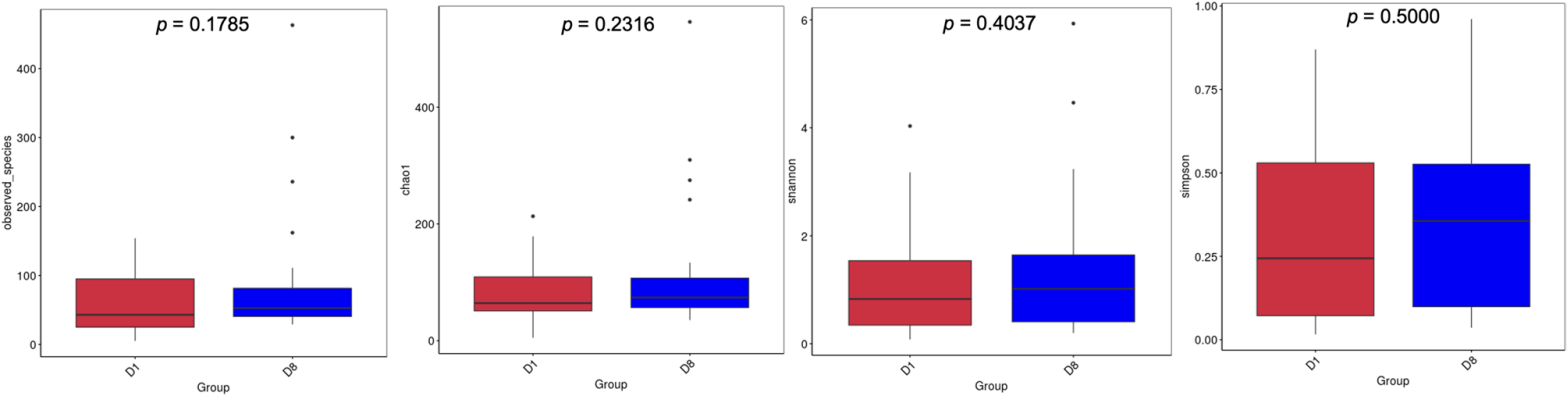
Boxplots of the alpha diversity indices (D1 and D8). Box plots showing observed species, as well as chao1- estimated richness and Shannon and Simpson diversity index for each group, calculated at the OTU level. These indices reflect the maximum, minimum, median and abnormal value of the index for each group. Each graph shows the *p*-value calculated using the Student t-test. Dots indicate the outliers

Alpha diversity describes the diversity within a sample. Rarefaction curves of the observed OTU numbers as a function of the sequence reads were calculated (Supplementary file: Fig. S2). The results of the comparison of the alpha diversity indices between groups D1 and D8 (number of species observed, Shannon, Chao1 and Simpson) can be seen in Fig. 4. No significant difference was found, revealing no difference in the richness and evenness within samples of the two groups. Group D8 showed slightly higher values in the Shannon diversity index and in the number of directly observed species (respectively, D1: 1.125; 62 and D8: 1.398; 90) (Fig. 4). Consequently, the samples in D8 show a more diverse and evenly distributed fungal community. The same trend was observed in the Chao1 index (estimation of the total number of species present) and Simpson’s index (estimation of diversity, like the Shannon index, but with greater weight for equability) (respectively, D1: 82.419; 0.315 and D8: 111.469; 0.363). The various alpha diversity indices analysis indicated that ICU setting did not have a significant impact on species richness.

**Fig. 5:**
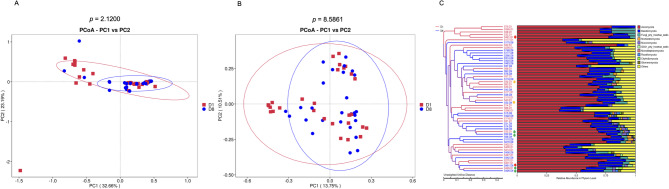
Beta diversity analysis. Representation of the fungal community of the two groups under study (D1 and D8). **A** Principal co-ordinate analysis (PCoA) of the fungi analysis of similarity by weighted UniFrac distance. **B** PCoA based on unweighted Unifrac distance. Each point represents a sample, represented by a principal component on the X axis and another principal component on the Y axis, which has been coloured by group. The percentage on each axis indicates the value of the contribution to the discrepancy between the samples. **C** Phylogenetic tree and phylum-level relative abundance map generated using the Unweighted Pair-Group Method with Arithmetic Mean (UPGMA) clustering based on Unweighted UniFrac distances. The dendrogram (left) shows the phylogenetic relatedness of skin mycobiomes at two time points (D1 in red, D8 in blue). The stacked bar plot (right) shows the phylum-level relative abundances. Green dots indicate patient pairs whose samples at D1and D8 clustered closely together

Beta diversity among samples was assessed by calculating both weighted (considers relative abundance of OTUs) or unweighted (presence/absence of OTUs) phylogenetic UniFrac distances at the OTU level. Principal coordinate analysis (PCoA) plot based on unweighted and weighted UniFrac dissimilarity revealed that it was not possible to identify a significantly different composition between the D1 and D8 groups (Fig. 5 A and B).

Phylogenetic analysis using the UPGMA tree (Fig. 5 C) revealed that, among the 21 patients with skin samples collected at both time points (D1 and D8), only two patient pairs (IDs 30 and 426) exhibited clear phylogenetic proximity between their D1 and D8 mycobiomes, as indicated by minimal branch distances in the dendrogram. These closely clustering pairs are highlighted with green dots in the figure for ease of identification. In contrast, the remaining 19 patient pairs (90%) showed greater phylogenetic divergence between time points, indicating that the skin-associated fungal communities underwent substantial changes during ICU hospitalization. This suggests that, although the overall genus-level composition appeared similar across patients, the underlying community structure is dynamic. Consistent with this, Adonis and ANOSIM analyses did not detect significant differences in overall community structure between groups (*p* = 0.260 and *p* = 0.499, respectively), supporting the notion that changes are patient-specific rather than systematic across the cohort.

### Comparison between culture-dependent and amplicon-based mycobiome profiling

To contrast the findings obtained through traditional culture methods with those derived from sequencing-based approaches, we evaluated the fungal community structure (mycobiome) in the subset of samples with known culture profiles (mycobiota). While culture-based methods identified *Candida* spp. exclusively in 48 out of 56 samples—primarily as single-species isolates—amplicon sequencing revealed a broader fungal diversity, including multiple *Candida* species per sample and non-*Candida* genera not captured by culture (Table 2).

Specifically, the metagenomic analysis uncovered the presence of filamentous and yeast-like genera such as *Penicillium*, *Cladosporium*, *Preussia*, *Thelephora*, *Clavispora*, *Aspergillus*, *Rhodotorula* and *Malassezia* in varying relative abundances (Fig. 2A). These taxa were not detected through routine culturing, highlighting the expanded resolution of high-throughput sequencing for fungal community characterization.

When comparing the relative abundances of the dominant *Candida* species between methods, *C. albicans* and *C. parapsilosis* remained predominant. Culture-based identification showed median proportions of 65.5% for *C. albicans* and 27.6% for *C. parapsilosis*, whereas amplicon sequencing revealed 34.2% and 33.3% median relative abundances, respectively.

Additionally, cohort stratification by sampling year (2021 vs. 2022) showed consistent relative abundance profiles for the predominant genera, confirming the reproducibility of both culture and sequencing findings across different time periods.

### Relationship between mycobiome and risk factors in ICU patients

The abundance of identified fungal species was also analysed for potential associations with the previously defined two patient groups evaluated at both admission (D1) and after eight days in the ICU (D8).

**Fig. 6:**
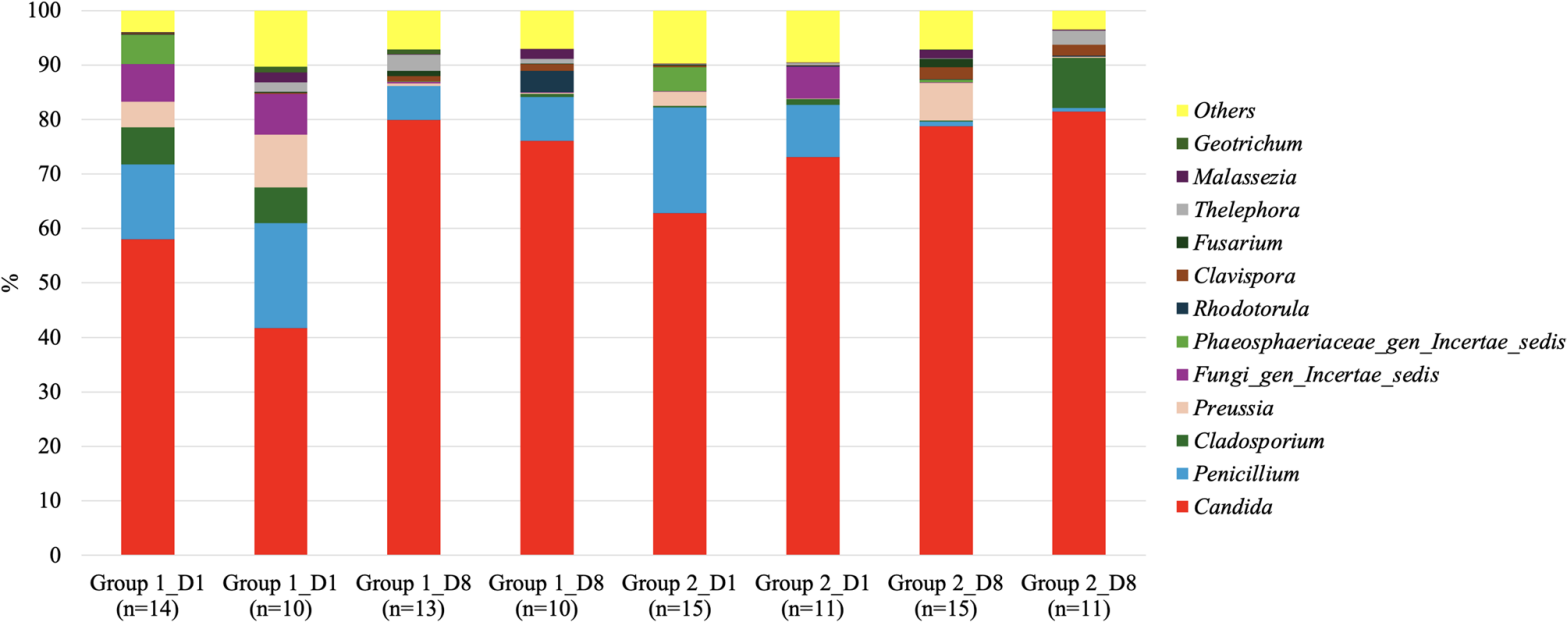
Relative abundance of fungal genera in axilla and inguinal swab samples. Bars show patients with one or two comorbidities and ICU risk factors (group 1_D1, 1_D8); paired patients with one or two comorbidities (group 1p_D1, 1p_D8); patients with more than two comorbidities and ICU risk factors (group 2_D1, 2_D8); and paired patients with more than two comorbidities (group 2p_D1, 2p_D8). Fungi are shown at genera level. Red bars indicate the total relative abundance of *Candida* sequences in each group relative to the total fungal sequences detected. “Others” represents the total relative abundance of species other than those named. By culturomics *Candida* spp. was detected in 12/14 (group 1D1), 9/13 (group 1D8), 14/15 (group 2D1) and 14/15 (group 2D8) patients. No other fungi were recovered by culture

Amplicon analysis of the skin niche in groups 1–2 at D1 and D8 (sequence threshold of 0.01% pooled) detected *Candida* spp. accounting for 58.0% and 62.8% of the total fungal genera in groups 1 and 2, respectively, at D1. At D8, *Candida* spp. accounted for 79.9% and 78.8% of the total fungal genera in groups 1 and 2, respectively (Fig. 6).

On average, the relative abundance of *Candida* spp. increased from D1 to D8 in both groups, with a concomitant decrease in species diversity. Importantly, paired samples within each group displayed minimal changes in community structure between D1 and D8, indicating a broadly stable mycobiome profile dominated by *Candida* regardless of patient comorbidity burden or ICU-specific risk factors. Importantly, paired samples within each group displayed minimal changes in community structure between D1 and D8, indicating a broadly stable mycobiome profile dominated by *Candida* regardless of patient comorbidity burden or ICU-specific risk factors. Although this increase did not reach statistical significance, it suggests that ICU-related conditions may promote *Candida* overgrowth, potentially contributing to an imbalance in the skin mycobiome.

Importantly, patients with only 1–2 comorbidities and ICU risk factors showed a degree of mycobiome disruption comparable to those with more than two comorbidities and intensive interventions. Notably, *Malassezia*, a commensal genus common in healthy skin, remained consistently low (below 0.5%) across all samples and timepoints, suggesting early and sustained disruption of the typical skin mycobiome upon ICU admission. These results indicate that the skin fungal community in critically ill patients is characterized by low diversity and marked *Candida* predominance with limited variation during the first week of intensive care.

## Discussion

Research on the cutaneous mycobiome has primarily focused on healthy individuals and its role in skin infections [6, 24]. Studies specifically investigating the skin mycobiome in ICU patients remain limited, although existing evidence suggests that fungal community composition can vary significantly in pathological conditions [16, 25].

In this pilot study, we observed a skin mycobiome with low diversity, strongly dominated by *Candida* spp. (69.3%) and a markedly low presence of *Malassezia* spp. This finding is consistent with previous reports of fungal imbalance in ICU settings, likely influenced by broad-spectrum antibiotics, immunosuppression, and invasive interventions [26, 27]. Comparable dysbiosis has also been described in the oral and respiratory mycobiomes of critically ill patients [28, 29].

Notably, our exploratory data suggest a possible competitive dynamic between *Candida* and *Malassezia*, where *Candida* may outcompete *Malassezia* under altered skin conditions such as inflammation or barrier disruption. This could be due to differences in nutrient utilization, as *Candida* can ferment carbohydrates and proliferate in glucose-rich, inflamed, or disrupted epithelial environments, while *Malassezia* depends on host-derived lipids and thrives in more stable, sebaceous conditions [2, 30].

In our cohort, which included predominantly older patients with comorbidities, colonisation by *C. albicans* (34.2%), and *C. parapsilosis* (33.2%) was frequent. Such colonisation likely reflects weakened skin barriers and altered immune responses. It is possible for the fungal species to compete through direct (e.g. nutrient availability) and/or indirect (e.g. antifungal immunity) mechanisms with the resident species of the mycobiome.

Direct comparisons with healthy individuals should be made cautiously due to differences in study design and patient factors. However, our preliminary observations raise the question of whether ICU patients harbour a more diverse range of non-*Malassezia* fungi than healthy controls—a hypothesis warranting investigation in studies with appropriate control groups and robust adjustment for confounders.

Our findings also highlight methodological challenges in mycobiome research, including variations in sampling, DNA extraction, and bioinformatics pipelines [31]. Standardizing these processes is essential for reliable inter-study comparisons. Consistent with earlier studies [32], our amplicon sequencing data aligned with culture results: *Candida* spp. were consistently detected by culture, while sequencing revealed additional fungi not recovered by culture alone. For example, genera such as *Penicillium*, *Cladosporium*, *Aspergillus* and *Malassezia* were detected only by sequencing, underscoring the limitations of conventional culture, which may overlook slow-growing or nutritionally demanding species.

It is important to interpret both culture and molecular findings with caution, as detection does not always imply true colonisation; transient environmental contamination is possible, particularly for ubiquitous fungi. This aligns with studies reporting higher fungal detection on exposed skin sites compared to covered areas [33]. In our cohort, transient detection of widespread fungi such as *Penicillium* and *Cladosporium* was most common at ICU admission, suggesting possible contamination. This highlights the need for rigorously controlled data analysis in human mycobiome research to ensure the reliability of sequencing results. These limitations can be addressed by analysing fungal communities (mycobiota) together with their genetic material (mycobiome), as we demonstrated in this pilot study.

Regarding changes over time, we found no significant differences in alpha or beta diversity between ICU admission (D1) and after one week (D8). There was a slight, non-significant increase in *Candida* relative abundance and a modest decline in overall fungal diversity. These trends may reflect the combined effects of ICU-related factors—such as antibiotic use, immunosuppression, and invasive procedures—that favour opportunistic fungi, while the controlled ICU environment, including air filtration and infection control protocols, may limit external microbial exposure and constrain major shifts [34].

Routine chlorhexidine gluconate (CHG) bathing, performed from D1 to D5, may also have influenced the mycobiome by temporarily reducing certain fungi. Previous work suggests that CHG effects on skin microbiota are transient, with recovery typically occurring within 24–72 h [35, 36]. Thus, while the D1 sample may have reflected recent CHG use, the D8 sample, collected at least 72 h after the last CHG bath, likely represents the skin mycobiome with minimal residual CHG impact.

Our study timeframe was chosen based on common ICU clinical protocols involving weekly assessments [37, 38]. Although we did not test a specific hypothesis regarding duration, this approach provides an initial perspective on short-term mycobiome dynamics. Future work should include longer follow-up to better capture temporal trends.

Although no significant changes were detected at the genus level, phylogenetic analyses suggest that the ICU environment may subtly reshape the fungal community structure, favouring certain phylogenetic lineages. This supports the notion that critically ill patients are at increased risk of colonisation by opportunistic fungi, particularly *Candida* spp [39, 40].

In an exploratory attempt to link clinical outcomes to mycobiome patterns, we observed that patients with longer ICU stays and multiple risk factors for *Candida* colonisation generally showed sustained dominance by *Candida* spp., consistent with prior studies [28, 29]. In this cohort, a poor outcome, defined as death within three months, was recorded for 52.9% of patients, although it is unclear whether these outcomes occurred in the hospital or post-discharge. Previous studies have established that critically ill patients with disrupted gut microbiota are at greater risk of poor outcomes and ICU-related complications [41]. However, it remains uncertain whether similar imbalances in microbial communities, such as the skin mycobiome, could have comparable impacts on patient outcomes.

In our study, the notable reduction of *Malassezia* and dominance of *Candida* spp. in ICU patients suggests a shift in the skin mycobiome toward a dysbiotic and potentially pathogenic state. This transition may be influenced by host-related factors (e.g., immune dysregulation, antibiotic exposure) and environmental pressures (e.g., antiseptic use, occlusion) and could increase susceptibility to fungal colonisation and infection during critical illness. In summary, our pilot study provides an initial characterization of the skin mycobiome in ICU patients, highlighting the predominance of *Candida* spp. and an apparent reduction of *Malassezia* in this setting.

However, several limitations must be acknowledged. Firstly, insufficient DNA quantities reduced the final sample size, limiting statistical power to detect associations with clinical factors. Of the 70 samples collected, DNA extraction and PCR amplification were successful in 56 (80%). The failure observed in the remaining 14 samples is likely attributable to the inherently low fungal biomass present on the skin, especially in critically ill patients frequently subjected to antiseptic procedures (namely CHG baths). Additionally, the presence of PCR inhibitors—such as skin lipids or components of the Amies transport medium—may have interfered with DNA yield or amplification efficiency.

We selected the axilla and groin as sampling sites given their heightened susceptibility to fungal colonisation in critically ill patients, ensuring both clinical applicability and practical feasibility in the ICU environment. While the study was limited to a single skin site and lacked an internal healthy control cohort, comparisons were drawn against established mycobiome data from published literature. Future research should include matched controls and sample multiple skin areas to better represent different skin microenvironments. Larger cohorts and extended monitoring beyond one week will help clarify temporal changes during longer ICU stays. Additionally, while culture and sequencing were complementary, standard mycological culture may underestimate the true diversity of fungi, missing slow-growing or nutritionally demanding species like *Malassezia* and certain moulds. Optimizing culture methods—such as using lipid-enriched media and extended incubation—alongside sequencing could improve detection. The routine use of antiseptics in ICU care may also influence fungal diversity, but this study did not assess this directly; future studies should compare mycobiome profiles before and after antiseptic exposure or under different disinfection protocols. Although the small sample size limits statistical significance and generalizability, this study was designed as an exploratory pilot to provide initial insights into the ICU skin mycobiome. The consistent predominance of *Candida* spp. highlights trends that warrant further investigation in larger, more diverse cohorts to validate these findings and explore their clinical relevance.

Finally, integrating bacterial and fungal microbiome analyses will be essential to understand their combined impact on colonisation dynamics and patient outcomes. Addressing these limitations will strengthen future investigations and help clarify the role of the skin mycobiome in critically ill patients.

## Conclusions

Our pilot study highlights that the ICU skin mycobiome in critically ill patients is characterized by low fungal diversity and a marked predominance of *Candida* spp., with only minor phylogenetic shifts observed during the first week of hospitalization. While the community structure appears generally stable under controlled ICU conditions, factors such as antimicrobial therapy, antiseptic bathing, and the patient’s underlying comorbidities likely contribute to subtle changes in fungal composition. These initial insights underscore the need for larger, well-controlled studies incorporating healthy comparators, multiple skin sites, extended monitoring, and standardized sequencing and culture methods. A deeper understanding of skin mycobiome dynamics in the ICU could clarify its role in colonisation, infection risk, and patient outcomes, and inform future infection control strategies.

## Supplementary information

Supplementary Material 1. Fig. S1: Electrophoretic Profile of PCR Products Amplified from ITS1 and ITS2 Regions. Electrophoretic profiles of PCR-amplified products using primers for the ITS1 and ITS2 regions, visualised on agarose gel stained with a fluorescent DNA marker (Green Safe). The images confirm the presence and quality of the amplified DNA, with bands corresponding to different molecular sizes. MW – molecular weight marker of 50–1500 bp, NZYDNA Ladder VI® (NZYTech, Lisbon, Portugal). Fig. S2. Alpha Diversity Rarefaction Curves Comparing Samples Groups D1 and D8. Rarefaction curves for alpha diversity indices (Simpson, Chao1, Observed Species, and Shannon) across sequencing depths for two samples groups (D1 and D8). Fig. S3. Principal Component Analysis (PCA) Plot of Sample Groups D1 and D8. This PCA plot visualizes the distribution of samples D1 (red squares) and D8 (blue circles) based on principal component analysis (PCA). PC1 (24.92%) and PC2 (18.59%) represent the first two principal components, capturing the highest variance in the dataset. Fig. S4. Principal Coordinates Analysis (PCoA) of Sample Groups. Principal Coordinates Analysis (PCoA) plots based on two principal coordinate axes (PC1 and PC2), representing variance in microbial community composition across multiple samples. Left Plot: PC1 (32.66%) and PC2 (23.19%) capture the largest variance in the dataset. Each point represents a sample, with different colors and shapes corresponding to unique sample IDs (D1 and D8). Right Plot: PC1 (13.75%) and PC2 (10.51%) capture a smaller proportion of variance compared to the left plot.

## Data Availability

The genome sequences generated in this study have been submitted to the NCBI SRA database (https://www.ncbi.nlm.nih.gov/sra/) under accession number PRJNA1159341.
