# Supplementary material for: Exploring the skin mycobiome in intensive care patients: a pilot study on fungal diversity from axillary and groin swabs
Source: BMC Microbiol. 2025 Sep 29;25:589. doi: 10.1186/s12866-025-04288-7 (PMC12482598; doi:10.1186/s12866-025-04288-7)
Supplement: Supplementary file 1 — Supplementary Material 1. Fig. S1: Electrophoretic Profile of PCR Products Amplified from ITS1 and ITS2 Regions. Electrophoretic profiles of PCR-amplified products using primers for the ITS1 and ITS2 regions, visualised on agarose gel stained with a fluorescent DNA marker (Green Safe). The images confirm the presence and quality of the amplified DNA, with bands corresponding to different molecular sizes. MW – molecular weight marker of 50–1500 bp, NZYDNA Ladder VI® (NZYTech, Lisbon, Portugal). Fig. S2. Alpha Diversity Rarefaction Curves Comparing Samples Groups D1 and D8. Rarefaction curves for alpha diversity indices (Simpson, Chao1, Observed Species, and Shannon) across sequencing depths for two samples groups (D1 and D8). Fig. S3. Principal Component Analysis (PCA) Plot of Sample Groups D1 and D8. This PCA plot visualizes the distribution of samples D1 (red squares) and D8 (blue circles) based on principal component analysis (PCA). PC1 (24.92%) and PC2 (18.59%) represent the first two principal components, capturing the highest variance in the dataset. Fig. S4. Principal Coordinates Analysis (PCoA) of Sample Groups. Principal Coordinates Analysis (PCoA) plots based on two principal coordinate axes (PC1 and PC2), representing variance in microbial community composition across multiple samples. Left Plot: PC1 (32.66%) and PC2 (23.19%) capture the largest variance in the dataset. Each point represents a sample, with different colors and shapes corresponding to unique sample IDs (D1 and D8). Right Plot: PC1 (13.75%) and PC2 (10.51%) capture a smaller proportion of variance compared to the left plot. [file 12866_2025_4288_MOESM1_ESM.docx]

Exploring the Skin Mycobiome in Intensive Care Patients: A Pilot Study on Fungal Diversity from Axillary/ groin swabs

Teresa Nascimento ^1*^, João Inácio ^2^, Daniela Guerreiro ^1^, Patrícia Patrício ^3^, Luís Proença ^1^, Cristina Toscano ^4^ and Helena Barroso ^1^

^1^ Department of Microbiology, Egas Moniz Center for Interdisciplinary Research (CiiEM), Egas Moniz School of Health & Science, Caparica, Almada, Portugal

^2^ School of Applied Sciences, University of Brighton, Brighton, United Kingdom

^3^ Department of Intensive Care, Hospital Beatriz Ângelo, Loures, Portugal

^4^ Department of Microbiology, Centro Hospitalar Lisboa Ocidental Hospital Egas Moniz, Lisboa, Portugal

* Corresponding Author

E-mail: tnascimento@egasmoniz.edu.pt (TN)

**Supplementary file**

**Fig. S1: Electrophoretic Profile of PCR Products Amplified from ITS1 and ITS2 Regions**

**Fig. S1** Electrophoretic profiles of PCR-amplified products using primers for the ITS1 and ITS2 regions, visualised on agarose gel stained with a fluorescent DNA marker (Green Safe). The images confirm the presence and quality of the amplified DNA, with bands corresponding to different molecular sizes. MW – molecular weight marker of 50–1500 bp, NZYDNA Ladder VI® (NZYTech, Lisbon, Portugal).

**Fig. S2: Alpha Diversity Rarefaction Curves Comparing Samples Groups D1 and D8**


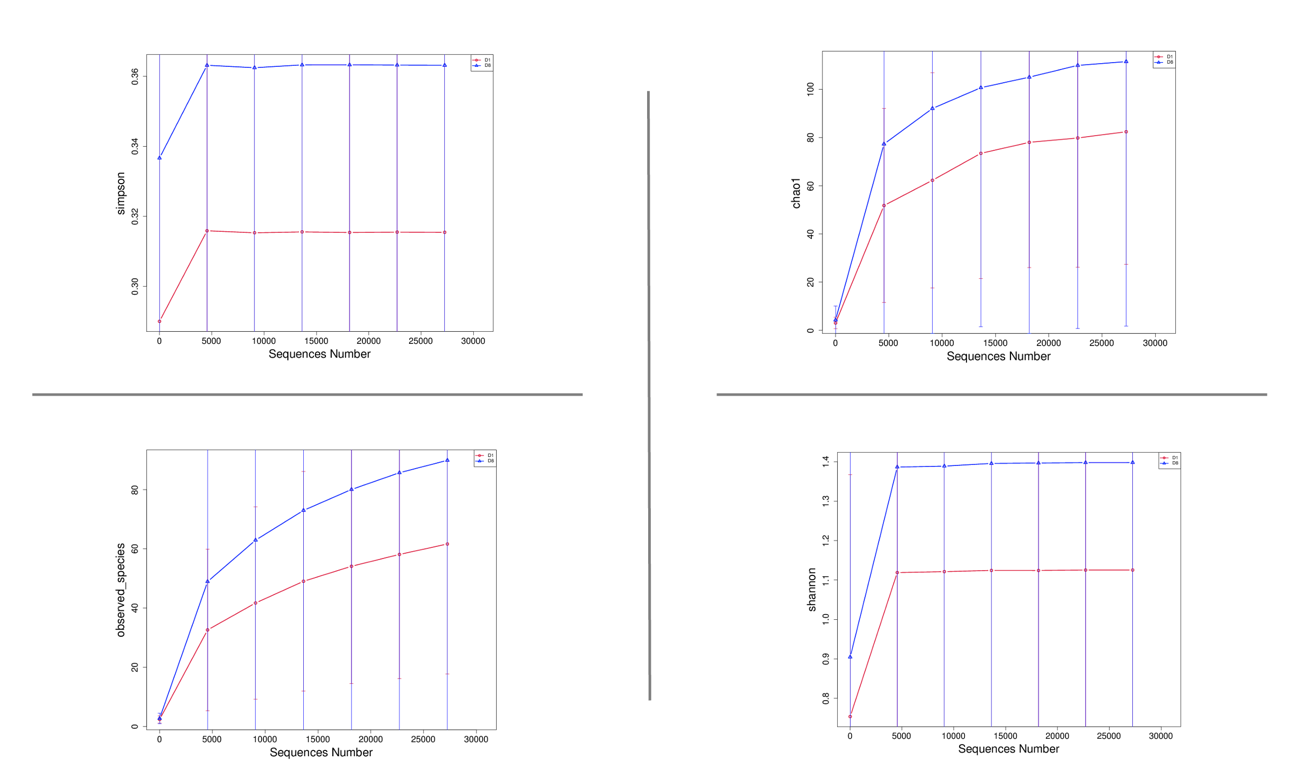


**Fig. S2** Rarefaction curves for alpha diversity indices (Simpson, Chao1, Observed Species, and Shannon) across sequencing depths for two samples groups (D1 and D8).

**
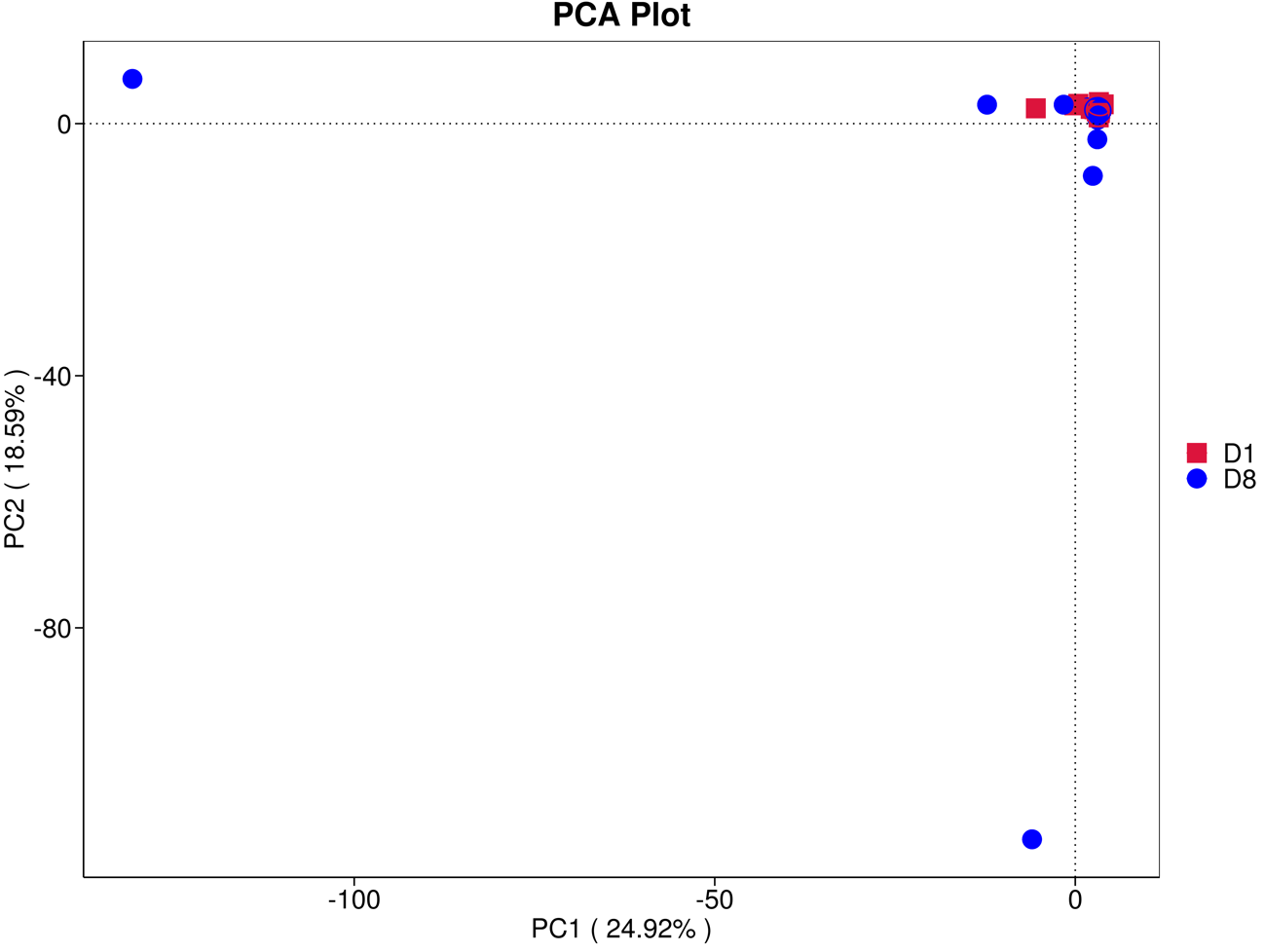
Fig. S3: Principal Component Analysis (PCA) Plot of Sample Groups D1 and D8**

**Fig. S3** This PCA plot visualizes the distribution of samples D1 (red squares) and D8 (blue circles) based on principal component analysis (PCA). PC1 (24.92%) and PC2 (18.59%) represent the first two principal components, capturing the highest variance in the dataset.

**Fig. S4 Principal Coordinates Analysis (PCoA) of Sample Groups**

**
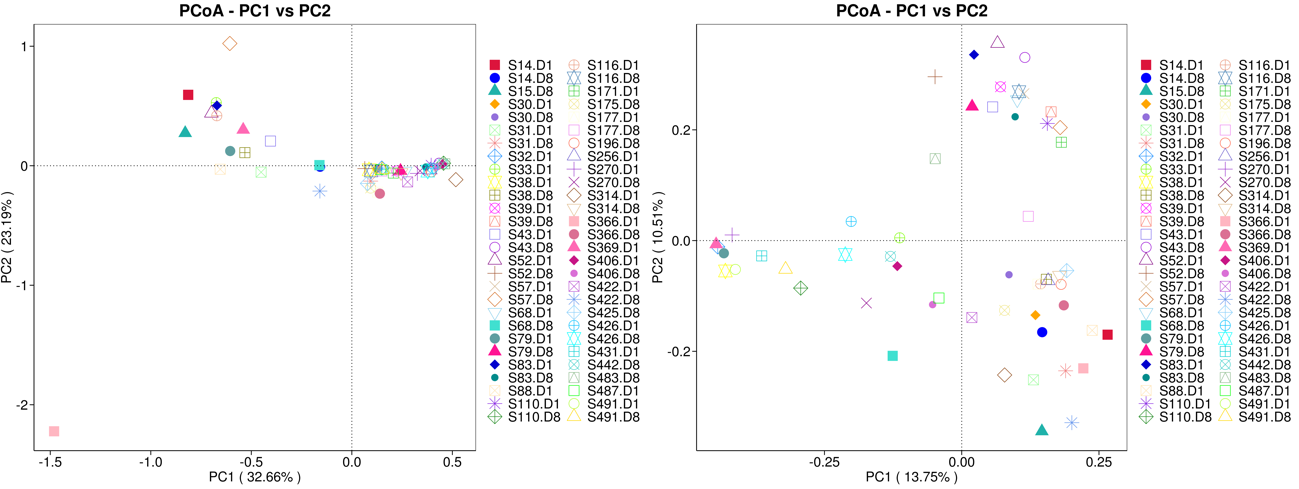
**

**Fig. S4** Principal Coordinates Analysis (PCoA) plots based on two principal coordinate axes (PC1 and PC2), representing variance in microbial community composition across multiple samples. Left Plot: PC1 (32.66%) and PC2 (23.19%) capture the largest variance in the dataset. Each point represents a sample, with different colours and shapes corresponding to unique sample IDs (D1 and D8). Right Plot: PC1 (13.75%) and PC2 (10.51%) capture a smaller proportion of variance compared to the left plot.
